# The Naturally Occurring Host Defense Peptide, LL-37, and Its Truncated Mimetics KE-18 and KR-12 Have Selected Biocidal and Antibiofilm Activities Against *Candida albicans*, *Staphylococcus aureus*, and *Escherichia coli In vitro*

**DOI:** 10.3389/fmicb.2017.00544

**Published:** 2017-03-31

**Authors:** Yu Luo, Denise T. F. McLean, Gerard J. Linden, Danny F. McAuley, Ronan McMullan, Fionnuala T. Lundy

**Affiliations:** ^1^Centre for Experimental Medicine, School of Medicine, Dentistry and Biomedical Sciences, Queen’s University BelfastBelfast, UK; ^2^Centre for Public Health, School of Medicine, Dentistry and Biomedical Sciences, Queen’s University BelfastBelfast, UK

**Keywords:** antimicrobial peptide, biofilm, human, LL-37, KE-18

## Abstract

Amongst the recognized classes of naturally occurring antimicrobials, human host defense peptides are an important group with an advantage (given their source) that they should be readily translatable to medicinal products. It is also plausible that truncated versions will display some of the biological activities of the parent peptide, with the benefit that they are less costly to synthesize using solid-phase chemistry. The host defense peptide, LL-37, and two truncated mimetics, KE-18 and KR-12, were tested for their inhibitory effects and antibiofilm properties against *Candida albicans*, *Staphylococcus aureus*, and *Escherichia coli*, microorganisms commonly implicated in biofilm-related infections such as ventilator-associated pneumonia (VAP). Using *in silico* prediction tools, the truncated peptides KE-18 and KR-12 were selected for minimum inhibitory concentration (MIC) and antibiofilm testing on the basis of their favorable cationicity, hydrophobic ratio, and amphipathicity compared with the parent peptide. Two methods were analyzed for determining peptide efficacy against biofilms; a crystal violet assay and an XTT [2,3-bis-(2-methoxy-4-nitro-5-sulfophenyl)-2H-tetrazolium-5-carboxanilide] assay. The biocidal activities (measured by MIC) and antibiofilm activities (measured by a crystal violet assay) appeared to be independent. LL-37 had no biocidal action against *C. albicans* (MIC > 250 μg/ml) but significant effects in both biofilm-prevention and biofilm-inhibition assays. KE-18 and KR-12 yielded superior MIC values against all three microorganisms. Only KE-18 had a significant effect in the biofilm-prevention assay, which persisted even at sub-MICs. Neither of the truncated peptides were active in the biofilm-inhibition assay. KE-18 was shown to bind lipopolysaccharide as effectively as LL-37 and to bind lipoteichoic acid more effectively. None of the peptides showed hemolytic activity against human erythrocytes at the concentrations tested. KE-18 should be considered for further development as a natural peptide-derived therapeutic for prevention of multi-species biofilm-related infections such as VAP.

## Introduction

The continual challenge to provide new approaches to combat antimicrobial resistance has prompted a resurgence of interest in non-conventional treatments for infection (Czaplewski et al., 2016). Novel therapeutic strategies are particularly relevant for medical device–related infections, such as ventilator-associated pneumonia (VAP) because of the potential for coating/treating devices (such as endotracheal tubes) with antimicrobials to prevent biofilm formation before insertion *in vivo*. Interventions of this type could, therefore, preclude pathogenic microorganisms becoming established in endotracheal tube biofilms, which are known to develop rapidly after intubation (Danin et al., 2014). The species diversity associated with VAP (Adair et al., 1999; Azoulay et al., 2006; Cairns et al., 2011) and the restricted potential for their eradication using conventional antibiotic treatments further emphasizes the need to investigate alternative therapeutic approaches for biofilm-prevention.

Antimicrobial peptides (AMPs) are naturally occurring antimicrobial agents, found in both the animal and plant kingdoms, with non-specific, immediate activities against a broad spectrum of microorganisms (Sang and Blecha, 2008). Several AMPs have been shown to have better antibiofilm activities than conventional antibiotics, owing to their rapid and broad-spectrum biocidal mechanisms, which appear to be effective against cells with low metabolic activities (Jorge et al., 2012). In addition, AMPs have also been shown to possess non-biocidal antibiofilm activities. For example, covalent immobilization of the AMP nisin onto a carbon-based surface was found to prevent the initial attachment of *Staphylococcus aureus* at concentrations below its minimal inhibitory concentration (MIC) (Qi et al., 2011). The 37-amino acid human cathelicidin, LL-37, has also been shown to have biofilm-prevention activities that are independent of its biocidal effects (Overhage et al., 2008).

In terms of generating novel peptides, both combinatorial (He et al., 2010) and rationally designed (Ashby et al., 2016) approaches have been used successfully to produce novel peptide libraries for biological screening. However, much remains to be learned about the exact nature of the structure-sequence-activity relationships that govern antimicrobial bioactivity, and thus, it continues to be challenging to design and generate functional AMPs *de novo*. Optimization of selected natural peptides, including peptide fragments and altered sequences based on natural AMPs, has facilitated discovery of novel short AMPs and improved their activity, providing excellent leads for therapeutic intervention. In view of this, our research group and others have used truncation of naturally occurring AMPs from human (Lundy et al., 2008), animal (Jittikoon et al., 2015), plant (Remuzgo et al., 2014), and bacterial (Zhou et al., 2015) sources as templates for the design of novel antimicrobials. Truncation of natural AMPs not only takes advantage of their evolutionary bioactivity but also advances them toward peptide therapeutics, which should ideally be short and compositionally simple, in order to minimize solid-phase synthesis costs.

Evidence from structure–activity relationship studies has demonstrated the importance of key characteristics such as peptide hydrophobicity and cationicity, but there are few comprehensive rules for natural peptide truncation, since the amino-terminal, mid-region, and carboxy-terminal sections of individual peptides may have variable structural features and resulting biological activities. Thus, it is important to utilize *in silico* prediction packages to guide peptide truncation in order to facilitate retention/enhancement of bioactivity with lower production costs. Several research groups have shown that the antibacterial activity of LL-37 resides in its mid-region (Li et al., 2006; Nell et al., 2006; Kanthawong et al., 2012) and thus truncation of this peptide could have particularly important cost benefits, given the expense of synthesizing a 37-mer peptide for therapeutic use. Stabilized LL-37 (D-enantiomer) as well as truncated mimetics of LL-37 have previously been shown to have antibiofilm activity against *Pseudomonas aeruginosa* (Dean et al., 2011; Nagant et al., 2012), but there is no information on the bioactivity and biofilm-prevention/inhibitory properties of LL-37 and truncated mimetics against other lung pathogens.

In the current work, we studied the bioactivity and biofilm-prevention/inhibitory properties of LL-37, and two truncated peptides, KE-18, and KR-12 against *Candida albicans*, *Staphylococcus aureus*, and *Escherichia coli.* All three microorganisms have recently been implicated in VAP (Hellyer et al., 2015), and *C. albicans* is of particular interest because fungal–bacterial interactions have the potential to modulate antibiotic efficacy (Harriott and Noverr, 2009, 2010), which could compromise conventional antibiotic treatment of multi-species infections.

In this study, truncation of LL-37 was shown to yield superior MIC values for KE-18 and KR-12 against all three microorganisms tested, but only KE-18 proved efficacious in the biofilm-prevention assay, and neither of the truncated peptides were active in the biofilm-inhibition assay. Furthermore, truncation was shown to retain or improve indirect antimicrobial activities such as lipopolysaccharide (LPS)- and lipoteichoic acid (LTA)-binding efficacy. Truncated mimetics of LL-37, and in particular KE-18, should thus be considered for prevention of multi-species biofilm-related infections such as VAP.

## Materials and Methods

### Microorganism Strains

*Escherichia coli* ATCC 25922 (LGC Standards, Middlesex, UK) and *S. aureus* NCTC 6571 (Public Health England, Salisbury, UK) were maintained on Colombia blood agar plates (Fannin, Galway, Ireland), and *C. albicans* NCTC 3179 was maintained on Sabouraud agar plates, prepared in-house using Sabouraud dextrose liquid media (Oxoid, Thermo Fisher, Hampshire, UK) and agarose (Helena Biosciences Europe, Tyne and Wear, UK).

### Peptides

LL-37 was supplied by Innovagen (Lund, Sweden). The truncated peptides KE-18 and KR-12 were custom synthesized by EZBiolab (Carmel, IN, USA) (**Table 1**).

**Table 1:** Amino acid sequences, overall net charge, and hydrophobic ratios of LL-37, KE-18, and KR-12 [charge and hydrophobic ratios were determined using the “calculation and prediction” feature of the Antimicrobial Peptide Database (http://aps.unmc.edu/AP/main.php)].

Peptide	Sequence	Charge	Molecular mass	Hydrophobic ratio
LL-37	LLGDFFRKSKEKIGKEFKRIVQRIKDFLRNLVPRTES	+6	4493.33	35%
KE-18	KEFKRIVQRIKDFLRNLV	+4	2302.80	44%
KR-12	KRIVQRIKDFLR	+4	1571.93	41%


### Radial-diffusion Assay for MIC Determination

A double-layer radial-diffusion assay was performed as previously described (McLean et al., 2013) to determine the MICs of LL-37, KE-18 and KR-12 against *C. albicans*, *S. aureus*, and *E. coli*. The underlay gel (10 ml) consisted of 1% (w/v) agarose containing approximately 4 × 10^5^ yeast cells or 5 × 10^6^ bacterial cells. Wells (2.5 mm in diameter) were punched in the agar, and 3 μl of peptide (0–250 μg/ml) was added prior to the addition of a nutrient-rich agarose over-layer (McLean et al., 2013). The plates were incubated for 18 h at 37°C and stained with a dilute solution of Coomassie brilliant blue R-250 as previously described (Lehrer et al., 1991; McLean et al., 2013). The antimicrobial activities were expressed as MICs, determined as the *x* intercept obtained from the relationship between radial-diffusion units versus log_10_ peptide concentration (Lehrer et al., 1991). The mean MICs were calculated from three replicate experiments.

### Inoculum Preparation for Biofilms

An overnight culture of *C. albicans* was grown in yeast extract peptone dextrose (YPD) broth (US Biological Life Sciences, Marblehead, MA, USA). Following centrifugation and washing in phosphate-buffered saline (PBS), the pellet was re-suspended in Roswell Park Memorial Institute (RPMI) 1640 medium (Cellgro Mediatech Inc., Manassas, VA, USA) and diluted to an optical density of 0.05 (600 nm; ∼1.0 × 10^6^ cells/ml) (Pierce et al., 2008). Inoculum preparations for *S. aureus* and *E. coli* were grown and diluted in brain-heart infusion (BHI) broth (Oxoid, Thermo Fisher, Hampshire, UK) to an optical density of 0.02 (600 nm; ∼5.0 × 10^6^ cells/ml) (Wang et al., 2007; Müsken et al., 2010).

### Biofilm-Prevention Assay

A biofilm-prevention assay was used to evaluate the ability of the peptides LL-37, KE-18, and KR-12 to prevent or reduce biofilm formation. Peptides were added to the inoculum preparations before addition to the wells of sterile, flat-bottomed, 96-well, microtiter plates (Nunc^®^, Sigma–Aldrich, Ayrshire, UK). Peptides were tested in the biofilm-prevention assay at either ×2 or at ×0.5 their MIC against the planktonic microorganism (**Table 2**). The only exception was LL-37, which was ineffective against *C. albicans* in the radial-diffusion assay, and thus, a concentration of twice the MIC of LL-37 against *S. aureus* was selected (i.e., 38.6 μg/ml; **Table 2**). Plates were then incubated at 37°C for 24 h to allow biofilm formation. Wells were washed three times with 200 μl PBS to facilitate removal of planktonic cells before quantification by the crystal violet assay or 2,3-bis-(2-methoxy-4-nitro-5-sulfophenyl)-2H-tetrazolium-5-carboxanilide (XTT) assay.

**Table 2:** Minimum inhibitory concentration (MIC) values of LL-37, KE-18, and KR-12 against *Candida albicans*, *Staphylococcus aureus*, and *Escherichia coli*.

Microorganism	MIC (μg/ml)		
	
	LL-37	KE-18	KR-12
*C. albicans*, NCTC 3179	>250	84 (±1)	5 (±2)
*S. aureus*, NCTC 6571	19.3 (±5)	7.2 (±0.6)	8.4 (±6.3)
*E. coli*, ATCC 25922	9.8 (±5.4)	2.1 (±1)	2.1 (±0.7)


*MIC values are the mean values (±SD) calculated from three individual radial-diffusion assays.*

### Biofilm-inhibition Assay

A biofilm-inhibition assay was used to evaluate the ability of LL-37, KE-18, and KR-12 to disrupt or inhibit the maturation of early biofilms. An overnight culture of microorganisms was prepared and diluted as described above. An initial biofilm was allowed to form for 4 hours, which has previously been shown to be sufficient for microbial attachment to plastic surfaces (Coenye et al., 2007; Pierce et al., 2008). Wells were then washed three times with 200 μl PBS to facilitate removal of planktonic cells before addition of peptides (at ×2 their MIC against each species except for LL-37 against *C. albicans*, as outlined above) in broth (100 μl). Plates were then incubated for further 24 h to allow biofilm maturation. Wells were washed, as outlined above, prior to biofilm quantification by the crystal violet assay or XTT assay.

### Crystal Violet Assay

The crystal violet assay was used to quantify the total biomass of biofilms, including cells and the extracellular matrix (Li et al., 2003). After the final washing step to remove planktonic cells, the biofilm was fixed with methanol and stained with crystal violet (Clin-Tech Ltd, Guildford, UK). Crystal violet dye that had bound to biofilms was released by adding 160 μl of 33% acetic acid to each well, and optical density was measured at 570 nm using a microtiter plate reader (GENios, Tecan, Männedorf, Switzerland).

### XTT Assay

XTT was used to measure the metabolic activity of cells within the biofilm. It was prepared as a saturated solution of 0.5 g/L in sterile RPMI, filter sterilized and stored at -70°C protected from light. 10 mM Menadione (Sigma-Aldrich, Ayrshire, UK) stock solution was prepared in 100% acetone and stored at -70°C. Menadione stock solution was added to XTT solution to make a XTT/menadione working solution containing 1 μM menadione; 100 μl XTT/menadione working solution was then added to prewashed biofilm well and negative control wells. Plates were incubated at 37°C for 2 h in the dark. A total of 80 μl of supernatant was then removed from each well for measurement of optical density at 490 nm (Pierce et al., 2008) using a microtiter plate reader (GENios, Tecan, Männedorf, Switzerland).

### Confocal Imaging of Biofilms

Biofilms for confocal imaging were grown on transparent ThinCert inserts (0.4 μm diameter) placed in six-well plates (Greiner Bio-One, Frickenhausen, Germany). KE-18 (×2 MIC) was added to the *S. aureus* inoculum preparations (1 ml in BHI, prepared as above) before addition to the ThinCert inserts. A further 1 ml BHI was also added to the bottom of wells during biofilm formation on the insert at 37°C for 24 h. The insert was washed three times with PBS to facilitate removal of planktonic cells and then stained with the LIVE/DEAD^®^BacLight Bacterial^TM^ Viability Kit, (Invitrogen, Carlsbad, CA, USA) according to the manufacturer’s instructions. The insert was then viewed by confocal laser scanning microscopy (LEICA-SP5 confocal with Leica Application Suite Advanced Fluorescence software) at ×100 magnification.

### LPS- and LTA-binding Assays

*Escherichia coli* LPS (Sigma–Aldrich, Ayrshire, UK) and *S. aureus* LTA (InvivoGen, San Diego, CA, USA) were biotinylated with biotin (long arm) hydrazide (Vector Laboratories, Peterborough, UK) using the biotin-labeling method outlined for glycoproteins as described by the manufacturer.

Peptide binding to biotinylated LPS was determined as previously described (McLean et al., 2013). Briefly, Greiner high-binding 96-well plates were coated with 100 μl of LL-37, KE-18, or KR-12 (6.25 μg/ml) in Voller’s buffer (26 mM Na_2_CO_3_, 23 mM NaHCO_3_, pH 9.6) overnight at 37°C. Plates were washed three times with Dulbecco’s PBS containing 0.05% Tween-20 (PBST) and blocked in PBST containing 1% (w/v) BSA for 1 h at room temperature. After washing three times with PBST, 100 μl of 1 ng/μl biotinylated *E. coli* LPS in PBS was added to each well and incubated at room temperature for 3 h with gentle agitation. Following three washes with PBST, detection of bound biotinylated LPS was achieved by adding 100 μl of streptavidin–horseradish peroxidase (BioLegend, London, UK), diluted 1:2000 in PBST for 30 min at room temperature. Following washing steps as above, peroxidase activity was detected with 2,2′-azino-bis(3-ethylbenzothiazoline-6-sulphonic acid) (ABTS; Invitrogen, Carlsbad, CA, USA; 100 μl/well) at 405 nm using a microtiter plate reader (GENios, Tecan, Männedorf, Switzerland).

Peptide binding to biotinylated LTA was determined as outlined above except that biotinylated LTA (1 ng/μl) was used in place of biotinylated LPS.

### Hemolytic Assay

The hemolytic assay was employed to determine potential peptide cytotoxicty against human erythrocytes (collected with ethical permission). Briefly, LL-37, KE-18, and KR-12 (0–175 μg/ml; 100 μl in PBS) were tested for their ability to release hemoglobin from an 8% suspension of fresh human erythrocytes (100 μl). One percent Triton X-100 (100 μl; Sigma–Aldrich) and 100 μl PBS were used as positive and negative controls, respectively. Samples and controls were incubated at 37°C for 1 h and then centrifuged for 5 min before reading the absorbance of the supernatant at 450 nm in a microtiter plate reader (Tecan GENios). Peptides were analyzed in triplicate in three independent assays. The % hemolysis was calculated using the equation:

$$\%\text{hemolysis = }\frac{\left({\text{Abs}}_{\text{sample}}-{\text{Abs}}_{\text{negative control}}\right)}{{\text{Abs}}_{\text{positive control}}-{\text{Abs}}_{\text{negative control}}}\times 100$$

### Statistical Analysis

Results for the antibiofilm properties of LL-37, KE-18, and KR-12 against *C. albicans*, *S. aureus*, and *E. coli* represent an average of three independent experiments and were subject to analysis by one-way ANOVA followed by Tukey’s *post hoc* correction for multiple comparisons. Results for the LPS- and LTA-binding efficacies of LL-37, KE-18, and KR-12 represent an average of three independent experiments and were also analyzed by one-way ANOVA followed by Tukey’s *post hoc* correction for multiple comparisons.

## Results

**FIGURE 1:**
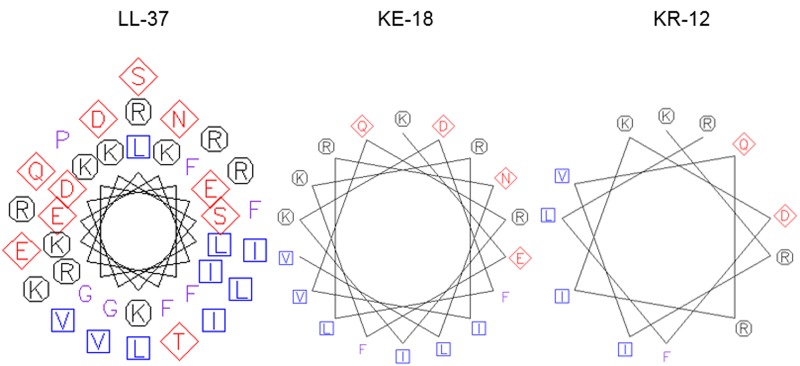
**Helical wheel projections of LL-37, KE-18, and KR-12.** Helical wheels were drawn using the EMBOSS pepwheel program (http://emboss.bioinformatics.nl/cgi-bin/emboss/pepwheel). Aliphatic residues (hydrophobic) are marked with squares, hydrophilic residues are marked with diamonds, positively charged residues are marked with octagons, and the remaining amino acids are unmarked. KE-18 and KR-12 show superior amphipathicity (hydrophobic residues confined to one face of the helix) compared with the parent LL-37 peptide.

Using *in silico* prediction software, truncation of LL-37 was shown to improve the hydrophobic ratio but decrease the charge of both KE-18 and KR-12 (**Table 1**). Helical wheel projection of LL-37, KE-18, and KR-12 showed that the truncated peptides displayed superior amphipathic helixes compared with the parent peptide (**Figure 1**). Truncation of LL-37 to KE-18 and KR-12 improved antimicrobial activity against *C. albicans*, *S. aureus*, and *E. coli*, as determined by radial-diffusion assays (**Table 2**).

**FIGURE 2:**
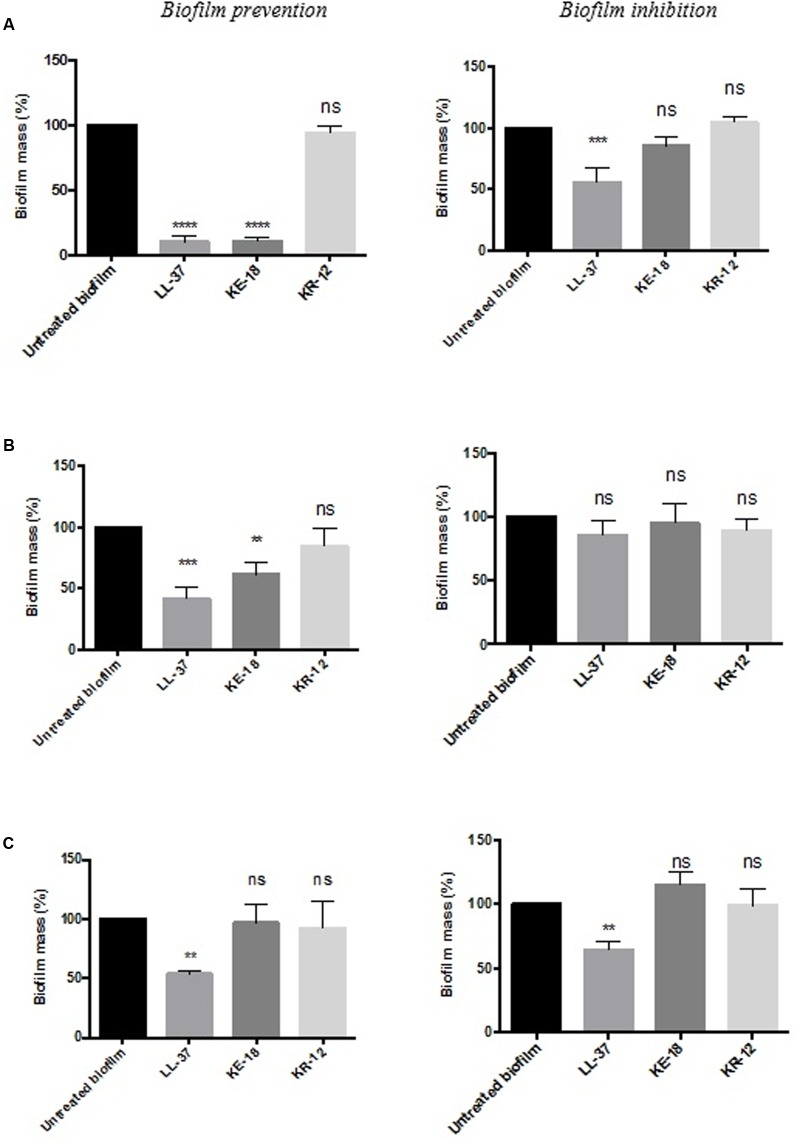
**Biofilm-prevention and biofilm-inhibition (of pre-formed biofilms) for LL-37, KE-18, and KR-12 against** **(A)** *Candida albicans*, **(B)** *Staphylococcus aureus*, and **(C)** *Escherichia coli*, measured using the crystal violet assay. One-way ANOVA followed by Tukey’s *post hoc* correction for multiple comparisons, *N* = 3 independent experiments, three replicates in each (^∗∗^*p* < 0.01; ^∗∗∗^*p* < 0.001; ^∗∗∗∗^*p* < 0.0001).

**FIGURE 3:**
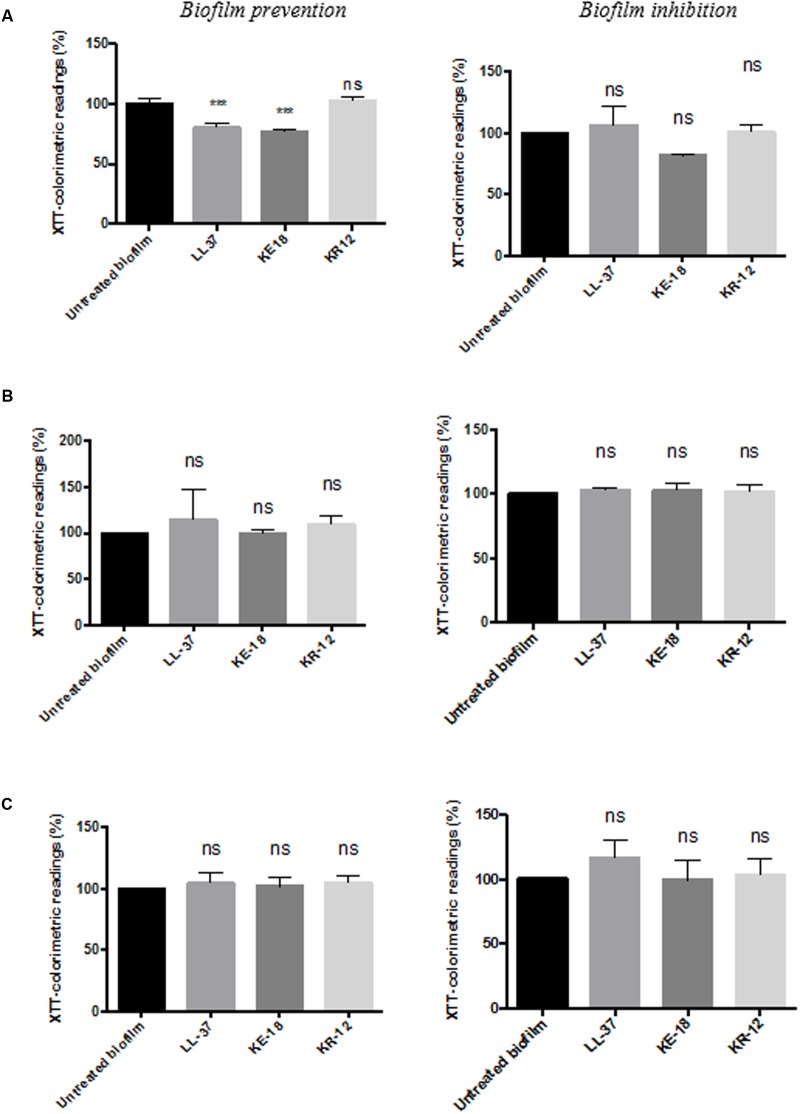
**Biofilm-prevention and biofilm-inhibition (of pre-formed biofilms) for LL-37, KE-18, and KR-12 against** **(A)** *C. albicans*, **(B)** *S. aureus*, and **(C)** *E. coli*, measured using the XTT assay. One-way ANOVA followed by Tukey’s *post hoc* correction for multiple comparisons, *N* = 3 independent experiments, three replicates in each (^∗∗∗^*p* < 0.001).

Quantification of biofilm biomass with the crystal violet assay (**Figure 2**) showed that LL-37 had significant efficacy in preventing biofilm formation by *C. albicans*, *S. aureus*, and *E. coli* and was also effective against early biofilms of *C. albicans* and *E. coli* but was unable to inhibit early biofilms of *S. aureus* (**Figure 2**). KE-18 showed significant activity against *C. albicans* and *S. aureus* in the biofilm-prevention assay but not in any of the biofilm-inhibition assays (**Figure 2**). KR-12 showed no antibiofilm properties against *C. albicans*, *S. aureus*, or *E. coli* in either biofilm-prevention or -inhibition assays (**Figure 2**). When the efficacy of the AMP family was investigated using the XTT metabolic assay (**Figure 3**), the results showed considerable differences to those reported with the crystal violet assay, with LL-37 and KE-18 preventing biofilm formation against *C. albicans* only (**Figure 3**).

**FIGURE 4:**
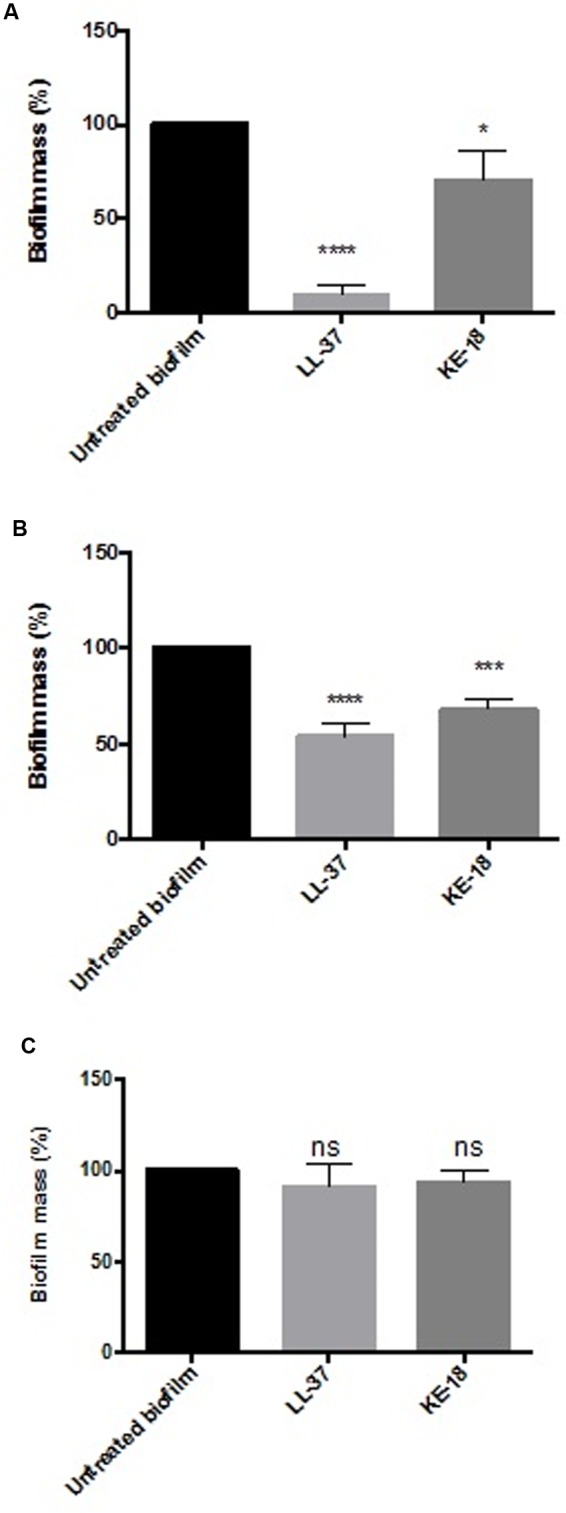
**Biofilm-prevention for LL-37 and KE-18 at sub-minimum inhibitory concentration (MIC) against** **(A)** *C. albicans*, **(B)** *S. aureus*, and **(C)** *E. coli*, measured using the crystal violet assay (^∗^*p* < 0.05; ^∗∗∗^*p* < 0.001; ^∗∗∗∗^*p* < 0.0001).

**FIGURE 5:**
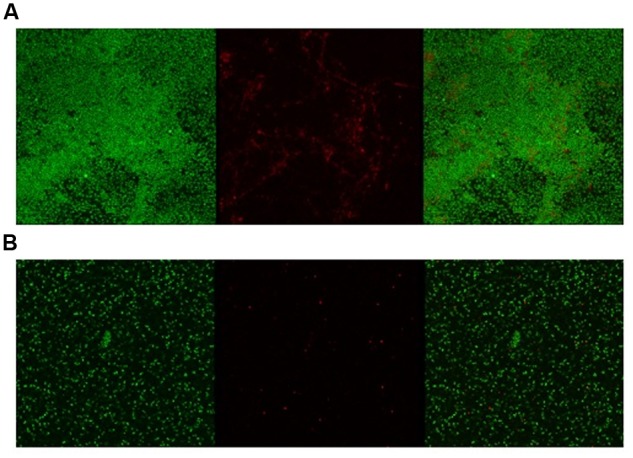
**Confocal imaging of live/dead-stained *S. aureus* biofilms [left, green channel (live); middle, red channel (dead); right, merged green and red channels].** **(A)** Untreated *S. aureus* biofilm and **(B)** KE-18-treated *S. aureus* biofilm.

**FIGURE 6:**
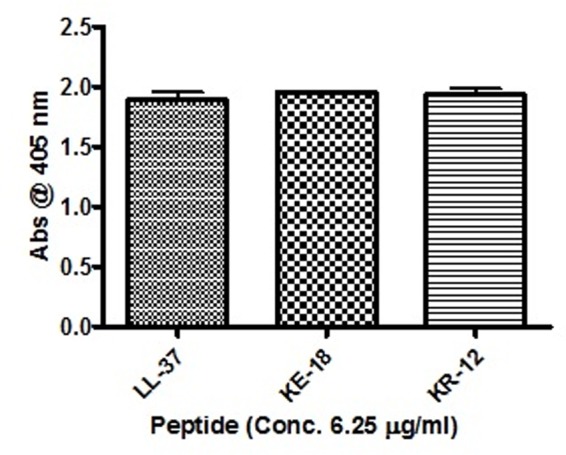
**Binding of LL-37, KE-18, and KR-12 to biotinylated *E. coli* lipopolysaccharide (LPS).** One-way ANOVA followed by Tukey’s *post hoc* correction for multiple comparisons showed no significant difference in LPS binding between LL-37 and its truncated mimetics.

**FIGURE 7:**
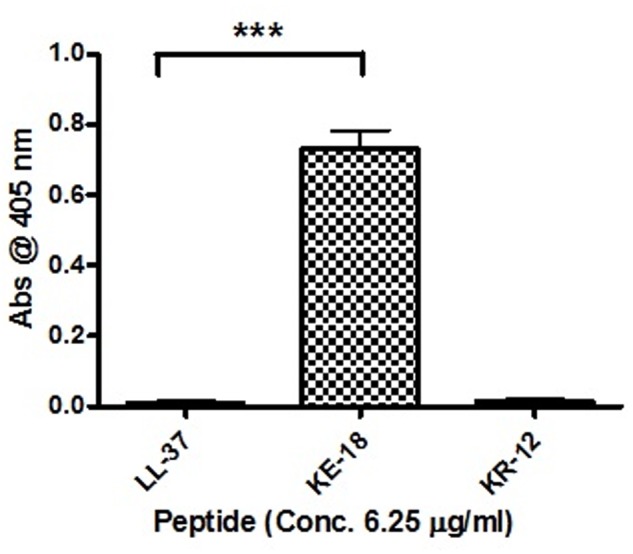
**Binding of LL-37, KE-18, and KR-12 to biotinylated *S. aureus* lipoteichoic acid (LTA).** One-way ANOVA followed by Tukey’s *post hoc* correction for multiple comparisons showed significantly enhanced LTA binding for KE-18 compared with LL-37 (^∗∗∗^*p* < 0.001).

Given the activity of LL-37 and KE-18 in biofilm-prevention, both peptides were also tested to determine their antibiofilm activities at sub-MICs (**Figure 4**). LL-37 and the truncated peptide KE-18 were shown to be efficacious against *C. albicans* and *S. aureus* biofilms at sub-MIC (**Figure 4**), but neither peptide displayed sub-MIC antibiofilm activity against *E. coli*. Confocal imaging of KE-18-treated biofilms (**Figure 5**) showed that KE-18 appeared to prevent attachment of *S. aureus* [as shown by a decrease in green staining (live)] following treatment rather than by direct killing [no increase in red staining (dead) with KE-18 treatment]. Both KE-18 and KR-12 were shown to retain the LPS-binding activity of LL-37 (**Figure 6**). Furthermore, KE-18 displayed significantly enhanced LTA-binding activity, while KR-12 retained the weak LTA-binding activity of the parent peptide (**Figure 7**). All peptides showed minimal activity in the hemolytic assay against human erythrocytes, even at the highest concentration of 175 μg/ml tested (**Table 3**).

**Table 3:** Hemolytic activities of LL-37, KE-18, and KR-12 against human erythrocytes.

Peptide/positive control	Concentration	Average % hemolysis
LL-37	175 μg/ml	4.47 (±0.35)
KE-18	175 μg/ml	1.17 (±0.18)
KR-12	175 μg/ml	0.45 (±0.10)
Triton X-100	1% (v/v)	100


*% hemolysis is the mean value (±SD) calculated from three individual hemolytic assays.*

## Discussion

In the current study, we focused on truncation of the naturally occurring AMP, LL-37, to KE-18 and KR-12 and showed that KE-18 retained planktonic and antibiofilm efficacy against the VAP-associated microorganisms *C. albicans*, *S. aureus*, and *E. coli*. The antimicrobial activity of the majority of AMPs is believed to depend on a combination of interdependent structural parameters including cationicity, hydrophobicity, and amphipathicity (Yeaman and Yount, 2003). In the current work, the reduction in cationicity compared with LL-37 may have been offset by a slightly higher hydrophobic ratio for KE-18 and KR-12. Additionally, it is recognized that amphipathicity (separation of charged groups from hydrophobic residues on the peptide) promotes interaction with negatively charged microbial membranes and subsequent penetration into the hydrophobic lipid bilayer (Fernandez-Vidal et al., 2007). The peptide amphipathicity of KE-18 and KR-12, visualized by helical wheel projection, revealed that the truncated peptides displayed superior amphipathic helixes to LL-37, which may have contributed to their enhanced antimicrobial activity, as determined by radial-diffusion assay.

In previous studies, LL-37 was found to possess unique antibiofilm activities [against *P. aeruginosa* (Overhage et al., 2008), *Staphylococcus epidermidis* (Hell et al., 2010), and *Francisella novicida* (Amer et al., 2010)]. However, only one study has previously focused on the antibiofilm activities of LL-37-derived peptides generated by truncation of both the *N*- and *C*-terminals (against *Acinetobacter baumannii*; Feng et al., 2013). Our observations on the *in vitro* efficacy of LL-37, KE-18, and KR-12 against *C. albicans*, *S. aureus*, and *E. coli* biofilms provide further support for the concept that the antibiofilm activities of AMPs are generally independent of their antimicrobial activities against planktonic cells (Overhage et al., 2008).

Several databases have been established to collate AMP sequences. The novel antibiofilm properties of LL-37, KE-18, and KR-12 reported here will be added to a recently established database specifically for biofilm-active AMPs (BaAMPs; Di Luca et al., 2015). Further investigation, however, will be required to determine which features of the LL-37 parent molecule specifically contribute to its antibiofilm activities. In particular, it will be of interest to determine whether the amphipathic helix portion of the parent molecule, needed for interaction with bacterial membranes (Wang et al., 2012), is necessary for antibiofilm activity. Interestingly, both KE-18 and KR-12 are devoid of the first six amino acids of LL-37, which have previously been shown to contribute to the cytotoxicity of the parent peptide (Nagant et al., 2012). In the current study, peptide hemolytic activity against human erythrocytes was tested up to a concentration of 175 μg/ml [so as to exceed the X2 MIC value for KE-18 against *C. albicans* (168 μg/ml)]. There was minimal hemolysis (<5%) of human erythrocytes even at the top concentration, and many of the peptides were active at much lower concentrations (<50 μg/ml), which could enhance their therapeutic potential.

The formation of biofilms on endotracheal tubes is considered a reservoir for respiratory pathogens in VAP patients (Pneumatikos et al., 2009), and thus, the prevention of biofilm formation by LL-37 and KE-18 could potentially serve to limit respiratory infections. Indeed, it is recognized that novel antibiofilm therapeutics need not fully eradicate biofilms but rather, by serving to reduce or delay their formation, they could allow secondary immune responses a better chance of combating potential infections (Singh et al., 2002). It has been previously reported that at sub-inhibitory concentrations, some conventional antibiotics (including aminoglycosides, fluoroquinolones, and tetracycline) can actually stimulate bacterial biofilm formation on medical devices (Hoffmann et al., 2005; Linares et al., 2006). Thus, it was important to show that LL-37, KE-18, and KR-12 had no such effects on the biofilms tested. In fact, both LL-37 and KE-18 continued to exhibit significant sub-MIC antibiofilm activity against *C. albicans* and *S. aureus*.

The antibiofilm properties of novel antimicrobials have previously been reported using both the crystal violet assay (Overhage et al., 2008) and the XTT assay (Martinez and Casadevall, 2006), and indeed, the use of more than one bioassay has recently been recommended (Ramage, 2016). It is acknowledged that assays utilizing different mechanisms for biofilm assessment may produce conflicting results. Furthermore, it is recognized that antimicrobial treatments can increase metabolic activity in the absence of increased biofilm as a result of protease secretion or the pumping out antimicrobials (Zasloff, 2002). Our results for the peptide-treated wells in the XTT assay concur with increased metabolic activity rather than increased cell numbers, since on visual inspection, peptide-treated biofilms in both the crystal violet and XTT assays appeared similar.

Given that naturally occurring peptides have important roles in innate host defense (Diamond et al., 2009), it is important to consider potential indirect effects such as their immunomodulatory actions. We showed that KE-18 and KR-12 retained the LPS-binding activity of LL-37 and, furthermore, that truncation of LL-37 to KE-18 significantly improved its ability to bind LTA. Thus, by effectively binding both LPS and LTA, KE-18 could be particularly important in reducing TLR-2 and TLR-4 stimulation, thereby limiting host pro-inflammatory responses.

In addition to its antimicrobial activities, LL-37 has emerged as both a positive and negative modulator of tumor growth/metastasis depending on the tumor type (Wu et al., 2010). *In vitro* studies have shown that the anticancer activity of LL-37 resides along with its antimicrobial activity in the central amphipathic helix (Li et al., 2006). Indeed, fragments of LL-37, including a 25 amino peptide, IG-25 (corresponding to amino acids 13–37 in the parent LL-37 peptide) showed anticancer activity against human cancer cell lines *in vitro* (Li et al., 2006). It is tempting to speculate that KE-18 and KR-12 may also exhibit anticancer activity given that their sequences fall within the IG-25 peptide; however, further work will be required to fully investigate this potential.

## Conclusion

In conclusion, we show, for the first time, the antimicrobial and antibiofilm properties of LL-37 and two truncated mimetics, KE-18 and KR-12, against the VAP-related microorganisms *C. albicans*, *S. aureus*, and *E. coli*. In light of the challenge of antimicrobial resistance, non-conventional treatments that may attenuate infection while conserving systemic antibiotics used for treatment, such as truncated AMPs, merit attention. In particular, KE-18 is promising in view of its favorable immunomodulatory and antibiofilm activities. Recent biofilm peptide immobilization approaches (Gao et al., 2011; Lim et al., 2015) offer exciting possibilities for the prevention of biofilm infections on medical devices and provide proof of concept for immobilizing antibiofilm peptides such as KE-18.

## Author Contributions

YL and DM undertook all the laboratory investigations; YL, DM, GL, RM, DM, and FL analyzed the data. YL drafted the manuscript and the co-authors reviewed and edited it. FL, GL, RM, and DM obtained the funding for this work.

## Conflict of Interest Statement

The authors declare that the research was conducted in the absence of any commercial or financial relationships that could be construed as a potential conflict of interest.
